# Cholecystectomy Is Linked With Lower Respiratory Exchange Ratio and Higher Lipid Oxidation and Sleep Energy Expenditure

**DOI:** 10.1002/oby.70145

**Published:** 2026-02-12

**Authors:** Beyza N. Aydin, Emma J. Stinson, Helen C. Looker, Peter Walter, Tomás Cabeza de Baca, Jonathan Krakoff, Douglas C. Chang

**Affiliations:** ^1^ Phoenix Epidemiology and Clinical Research Branch National Institute of Diabetes and Digestive and Kidney Diseases Phoenix Arizona USA; ^2^ Clinical Mass Spectrometry Core National Institute of Diabetes and Digestive and Kidney Diseases Bethesda Maryland USA

**Keywords:** gallbladder, Indigenous American, metabolic chamber, respiratory quotient, whole room indirect calorimetry

## Abstract

**Objective:**

Cholecystectomy (GBX) may alter energy metabolism, but human evidence is limited. We examined whether GBX alters energy expenditure (EE), respiratory exchange ratio (RER), and substrate oxidation.

**Methods:**

A total of 384 healthy Southwestern Indigenous American adults (222 males, age 28 ± 6 years) were studied, including individuals with a history of gallbladder surgery [GBX(+), *n* = 39] and without surgery [GBX(−), *n* = 345]. In addition, 24‐h energy metabolism was measured in a respiratory chamber. General linear models were adjusted for age, sex, body composition, and glucose regulation. RER and macronutrient oxidation rates were further adjusted for energy balance.

**Results:**

GBX(+) participants were older (31 ± 7 vs. 27 ± 6 years, *p* = 0.0002) and mostly female (95% vs. 36%, *p* < 0.0001), and they had higher body fat (40% ± 5% vs. 32% ± 8%, *p* < 0.0001), although body composition differences were sex related. Adjusted models showed lower RER (*β* = −0.01, *p* = 0.01), higher lipid oxidation (*β* = 79 kcal/day, *p* = 0.03), and higher sleep EE (*β* = 78 kcal/day, *p* = 0.006) in the GBX(+) group. Other EE variables and macronutrient oxidation rates were not significantly associated with GBX history (all *p*'s > 0.1).

**Conclusions:**

Independent of obesity, an absent gallbladder is associated with decreased RER and increased lipid oxidation and sleep EE rates, indicating that the gallbladder may have a role in metabolic fuel selection that has implications for metabolic health.

**Trial Registration:**

ClinicalTrials.gov identifiers: NCT00339482, NCT00340132

## Study Importance

- What is already known?
  ○ Cholecystectomy (GBX) alters bile acid circulation and may impact energy and glucose metabolism.
  ○ Animal studies and limited human data suggest possible changes in energy expenditure after GBX.
- What does this study add?
  ○ Prior GBX is associated with increased sleep energy expenditure, higher lipid oxidation, and lower respiratory exchange ratio.
  ○ These effects are independent of body composition, age, sex, and glucose regulation status
- How might these results change the direction of research or the focus of clinical practice?
  ○ GBX may have lasting effects on energy metabolism and substrate utilization, highlighting the need to consider metabolic outcomes when deciding on surgery and managing patients post surgery.

## 1 Introduction

Cholecystectomy (GBX), the surgical removal of the gallbladder, is considered a safe and low‐risk procedure and is one of the most frequently performed surgical procedures primarily to treat gallstones [1]. Gallstone disease is common worldwide but disproportionately affects Indigenous [2] and Mexican American [3] populations compared to White individuals.

In the absence of a gallbladder after GBX, bile flows more rapidly and undergoes more frequent cycling between the liver and intestine (enterohepatic circulation) in both animal models and humans [4, 5, 6, 7]. This enhances exposure to bile acids (BAs) particularly during fasting, as rhythmic episodes of postprandial gallbladder emptying and refilling are disrupted [8]. Beyond its impact on bile storage, removal of the gallbladder may have potential consequences for energy metabolism that are not completely understood [9].

Primary BAs include cholic acid and chenodeoxycholic acid and are the immediate products of BA synthetic pathways. After synthesis, BAs may be conjugated with taurine or glycine. Secondary BAs are produced via bacterial modifications of primary BAs in the intestines. Besides their long recognized role in facilitation of lipid absorption, it is now known that BAs function as hormones regulating lipid, glucose, and energy metabolism [8] through the nuclear receptor farnesoid X receptor (FXR) and the G protein‐coupled BA receptor 1 (GPBAR‐1 or TGR5) [10]. Through an agonist effect on TGR5, BAs promote energy expenditure (EE) in mice by inducing intracellular thyroid hormone activation independently of FXR‐α [11]. In addition, there are some studies suggesting that GBX may lead to metabolic consequences, including changes in glucose, insulin, lipid, and lipoprotein levels, hepatic steatosis, and metabolic syndrome [8, 12]. Furthermore, the increased exposure of BAs to intestinal bacteria following GBX can alter their composition [8]. In a mouse model, Cortes et al. reported increased EE following GBX [13]; however, evidence that long‐term human EE is altered after GBX is limited [14].

The current study investigated whether participants without gallbladders after GBX [referred to as GBX(+)] have altered EE, respiratory exchange ratio (RER), and substrate oxidation in comparison to participants with no history of gallbladder removal [referred to as GBX(−)]. In a cohort of Indigenous Americans in the Southwestern United States, volunteers with and without prior GBX had measurements completed over 24 h in a whole‐room indirect calorimeter.

## 2 Methods

### 2.1 Study Design

This analysis included participants of Indigenous American descent who were enrolled in a longitudinal cohort study in the Southwestern United States (NCT00339482) [15]. A subset of participants in this study agreed to participate as adults in an inpatient study with detailed metabolic phenotyping examining risk factors for obesity, diabetes, and related complications (NCT00340132) [16], including measurement of EE. Both studies were approved by the Institutional Review Board of the National Institutes of Health and the participating tribe. Written informed consent was obtained from all volunteers.

During the inpatient stay at the clinical research unit in Phoenix, Arizona (1985–2007), participants were screened by medical history, physical examination, and routine screening laboratory tests to ensure that they were healthy. Prior GBX history was ascertained through medical history and chart review. None of the participants was taking medication that could affect energy or glucose metabolism. The women underwent a urine pregnancy test to ensure that they were not pregnant. After screening, participants were enrolled in the study and placed on a weight‐maintaining diet (WMEN) (50% carbohydrate, 30% fat, and 20% protein; food quotient of 0.87) based on sex and weight, as previously described [17]. Because the standardized run‐in diet provided 50% carbohydrate, prior work indicates that adaptation of 24‐h respiratory quotient to this macronutrient pattern is relatively rapid (about 1–2 days to reach 50% of the expected change) [18]. Therefore, a 5‐day standardization is likely sufficient to limit dietary carry‐over effects, although residual influences from habitual intake cannot be completely excluded. After at least 3 days on this diet, a 3‐h 75‐g oral glucose tolerance test (OGTT) was performed to confirm that participants did not have diabetes according to the 2003 American Diabetes Association criteria (fasting plasma glucose < 126 mg/dL and 2‐h plasma glucose < 200 mg/dL) [19]. Fasting blood drawn during the OGTT was used to process for BAs. During this inpatient stay, body composition measurement and, after a minimum 5‐day WMEN diet, a 24‐h respiratory chamber stay were also performed.

### 2.2 Body Composition

Body composition was measured by hydrodensitometry or dual‐energy X‐ray absorptiometry (DPX‐L and Prodigy; GE/Lunar Co.) [20]. Absorptiometric measurements were converged to comparable hydrodensitometry values as previously described [21, 22].

### 2.3 Respiratory Chamber

Twenty‐four–hour EE, RER, and spontaneous physical activity (SPA) were measured in a whole‐room indirect calorimeter, as previously described [23]. The prescribed energy intake during the chamber stay included four balanced meals and was reduced by approximately 80% during these periods of restricted physical activity. Twenty‐four–hour energy intake was calculated using the following formulas: Males: energy intake (kcal) = 1238 + (14.1 × body weight in kg) − (5.7 × body mass index [BMI]); Females: energy intake (kcal) = 951 + (19.3 × body weight in kg) − (18.2 × BMI) [24]. Only participants whose energy balance (24‐h energy intake minus 24‐h EE) was within ±20% were included in the analysis sample. Carbon dioxide production, oxygen consumption, RER, and EE rate were measured and calculated using Lusk's equation [25]. Twenty‐four–hour rates of carbohydrate oxidation (CarbOx) and lipid oxidation (LipOx) were calculated based on the 24‐h RER, accounting for protein oxidation (ProtOx), which was estimated from 24‐h urinary nitrogen excretion, as previously reported [26, 27]. EE was recorded continuously, computed in 15‐min intervals, averaged, and projected over 24 h. SPA was assessed using two Doppler‐based microwave radar sensors placed within the respiratory chamber to detect movement and determine the percentage of time participants were physically active [23]. A detailed description of this method has been published previously [28]. Sleep EE was determined by averaging EE values recorded between 1:00 and 5:00 a.m., during periods when SPA was below 1.5% (< 0.9 s/min), and then extrapolated to represent 24 h [29]. Inactive state EE (EE_0_) was defined as the intercept of the regression line plotting EE against SPA during the period from 11:00 to 1:00 a.m. [23]. Awake and fed thermogenesis (AFT), representing EE associated with being awake and in the fed state (i.e., thermic effect of food), was calculated as the difference between EE_0_ and sleep EE.

### 2.4 Analytical Measures

Individual BA detection and quantification were performed by UPLC‐MS/MS using a Thermo Scientific Vanquish UPLC, coupled with a Thermo Scientific ALTIS triple quadrupole mass spectrometer and heated electrospray ionization (HESI‐II) in negative ion mode (2500 V). Further details are provided elsewhere [30].

### 2.5 Statistical Analyses

Statistical analyses were performed using SAS, version 9.4 (SAS Institute Inc.). Baseline data of GBX(+) participants were compared with GBX(−) (reference group). Alpha was set at 0.05 and two‐sided *p*‐values were reported. Normally distributed data are reported as mean ± SD. Undetectable BA concentrations (< 0.25 ng/mL) were assigned a value of 0.125 ng/mL (half the detection limit) for analysis. Of all the BAs analyzed, there were five BAs with this assigned value: HDCA (*n* = 1), LCA (*n* = 1), TDCA (*n* = 1), TLCA (*n* = 8), and UDCA (*n* = 3). Due to their skewed distribution, values were log_10_‐transformed for analysis.

Independent sample *t*‐tests for continuous variables or chi‐square tests for categorical variables were used for group comparisons [GBX(+) vs. GBX(−)]. General linear models (GLM) were used to investigate group differences [GBX(+) vs. GBX(−)] on measures of energy metabolism. All models were adjusted for age, sex, body composition, and impaired glucose regulation (vs. normal glucose regulation). RER and macronutrient oxidation rates were further adjusted for energy balance during the eucaloric chamber. Figures presenting results display adjusted (i.e., residual) values consistent with the covariates used in the models. Mean values were added back to residual values to restore the original scale. Sensitivity analyses were conducted in subgroups of females and individuals with normal glucose regulation.

## 3 Results

### 3.1 Participant Characteristics

Of the 834 participants who had EE measured, 450 volunteers were excluded due to invalid calorimetry (*n* = 40), uncertain history of GBX (*n* = 374), OGTT indicating diabetes (*n* = 28), and missing OGTT results (*n* = 5) (Figure S1). Thus, 384 persons were analyzed (345 with and 39 without prior GBX). Characteristics of these 384 participants are presented in Table 1. Compared to GBX(−), GBX(+) participants were older (31.3 ± 6.6 vs. 27.3 ± 6.3 years, *p* = 0.0002) and more likely to be female (95% vs. 36%, *p* < 0.0001) and have higher BMI (38.1 ± 9.5 vs. 34.0 ± 8.2 kg/m^2^, *p* = 0.004) and body fat percentage (39.5 ± 5.5 vs. 32.1 ± 8.1, *p* < 0.0001) although body composition differences were explained by sex (Table S1).

**TABLE 1:** Characteristics of participants with (+) and without (−) prior GBX.

Variable	Total	GBX(+)	GBX(−)	*p*
*n* (%)	384 (100)	39 (10)	345 (90)	
Demographics				
Age (years)	27.7 ± 6.4	31.3 ± 6.6	27.3 ± 6.3	0.0002
Sex, *n* (%)				< 0.0001
Female	162 (42)	37 (23)	125 (77)	
Male	222 (58)	2 (1)	220 (99)	
Body composition				
Weight (kg)	95.7 ± 24.8	98.2 ± 27.6	95.4 ± 24.5	0.5
BMI (kg/m^2^)	34.4 ± 8.4	38.1 ± 9.5	34.0 ± 8.2	0.004
Body fat (%)	32.8 ± 8.2	39.5 ± 5.5	32.1 ± 8.1	< 0.0001
Fat mass (kg)	32.5 ± 14.6	39.7 ± 16.0	31.7 ± 14.2	0.001
Fat‐free mass (kg)	63.1 ± 13.3	58.5 ± 12.9	63.7 ± 13.2	0.02
Oral glucose tolerance test				
Fasting plasma glucose (mg/dL)	88.9 ± 9.7	93.7 ± 9.8	88.3 ± 9.6	0.001
2‐h plasma glucose (mg/dL)	122.4 ± 30.5	143.4 ± 26.8	120.0 ± 30.0	< 0.0001
Glucose regulation status, *n* (%)				0.0005
Normal glucose regulation	254 (66)	16 (6)	238 (94)	
Impaired glucose regulation	130 (34)	23 (18)	107 (82)	

*Note*: Values are expressed as mean ± SD or *n* (%). *p*‐values for differences between GBX(+) and GBX(−) by *t*‐test or chi‐square test where appropriate.

Abbreviation: GBX, cholecystectomy.

### 3.2 Energy Expenditure and Spontaneous Physical Activity

**FIGURE 1:**
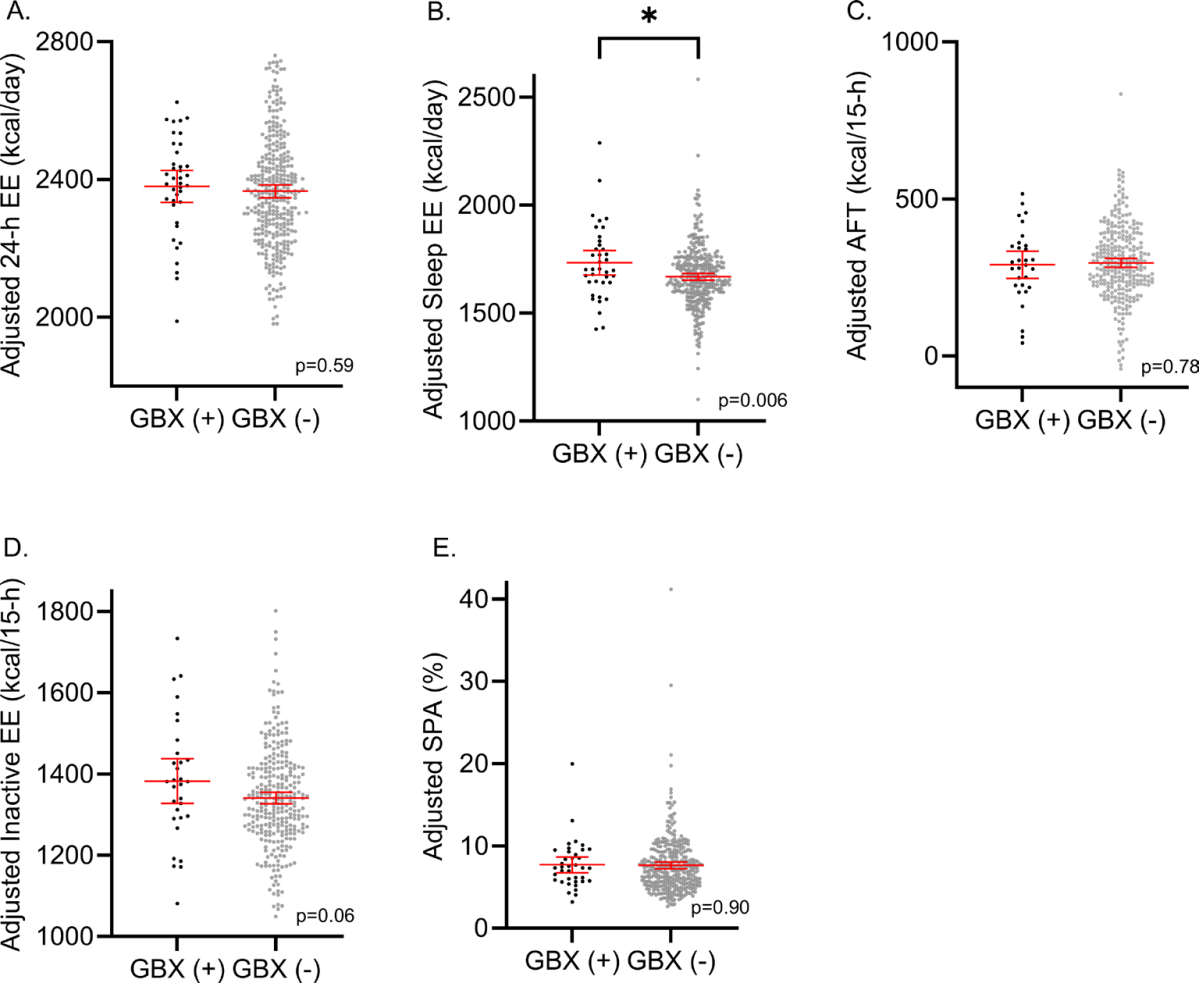
Differences in (A) 24‐h energy expenditure (EE), (B) sleep EE, (C) awake and fed thermogenesis (AFT), (D) inactive state EE, and (E) spontaneous physical activity (SPA) between those with and without prior cholecystectomy (GBX). Sleep EE was higher in the cholecystectomy group. Models were adjusted for age, sex, body composition, and impaired glucose regulation (vs. normal glucose regulation). Mean values were added back to residuals to restore the original scale. Asterisk (*) denotes statistical significance (*p* < 0.05). [Color figure can be viewed at wileyonlinelibrary.com]

Twenty‐four‐hour sleep EE (*β* = 5.60 kcal/day, SE = 54.02, *p* = 0.92), AFT (*β* = −43.20, SE = 23.77, *p* = 0.07), inactive state EE (*β* = −41.25, SE = 44.13, *p* = 0.35), and SPA (*β* = −0.93, SE = 0.62, *p* = 0.13) did not differ between GBX(−) and GBX(+) in unadjusted models (Table 2). Twenty‐four‐hour EE was significantly lower in GBX(+) (*β* = −155 kcal/day, SE = 71.02, *p* = 0.03), but there was no significant difference after adjustment for covariates (*β* = 17.20 kcal/day, SE = 32.16, *p* = 0.59, Figure 1A). In the adjusted analysis, sleep EE was higher in GBX(+) (*β* = 78 kcal/day, SE = 28.11, *p* = 0.006, Figure 1B; see Figure S2 for scatterplot of adjusted 24‐h sleep EE and fat‐free mass). In separate adjusted sensitivity analyses in the female subgroup and in the subgroup with normal glucose regulation, results were similar (Figures S3B and S4B, respectively). AFT (*β* = −7.20 kcal/15‐h, SE = 25.74, *p* = 0.78), inactive EE (*β* = 50.89 kcal/15‐h, SE = 26.64, *p* = 0.06), and SPA (*β* = 0.08%, SE = 0.68, *p* = 0.90) did not differ between GBX(−) and GBX(+) in adjusted models (Figure 1C–E). In a matched case–control sensitivity analysis, a similar pattern of findings emerged (EE, RER, and substrate oxidation results in Table S3).

**TABLE 2:** Calorimetry results of participants with (+) and without (−) prior GBX.

Variable	All	GBX(+)	GBX(−)	*p*
24‐h EE (kcal/day)*	2368 ± 422	2229 ± 407	2384 ± 422	0.03
24‐h sleep EE (kcal/day)	1676 ± 315	1681 ± 357	1675 ± 310	0.9
Awake and fed thermogenesis (kcal/15‐h)	296 ± 124	257 ± 112	300 ± 125	0.07
Inactive state EE (kcal/15‐h)	1346 ± 234	1308 ± 261	1350 ± 231	0.4
Spontaneous physical activity (%)	7.65 ± 3.62	6.82 ± 2.84	7.75 ± 3.69	0.1
24‐h RER (ratio)*	0.850 ± 0.023	0.841 ± 0.027	0.851 ± 0.022	0.02
24‐h Non‐protein RER (ratio)*	0.858 ± 0.027	0.849 ± 0.035	0.859 ± 0.026	0.02
24‐h Lipid oxidation rate (kcal/day)	963 ± 295	967 ± 310	963 ± 294	0.9
24‐h Carbohydrate oxidation rate (kcal/day)*	1083 ± 238	960 ± 259	1097 ± 231	0.0006
24‐h Protein oxidation rate (kcal/day)	297 ± 114	276 ± 109	300 ± 114	0.2
24‐h Energy balance (kcal/day)	−91.7 ± 182	−68.3 ± 159	−94.3 ± 185	0.4

*Note*: Values are expressed as mean ± SD. *p*‐values for differences between GBX(+) and GBX(−) by *t*‐test.

Abbreviations: EE, energy expenditure; GBX, cholecystectomy; RER, respiratory exchange ratio.

* *p* < 0.05.

### 3.3 Respiratory Exchange Ratio and Substrate Oxidation

**FIGURE 2:**
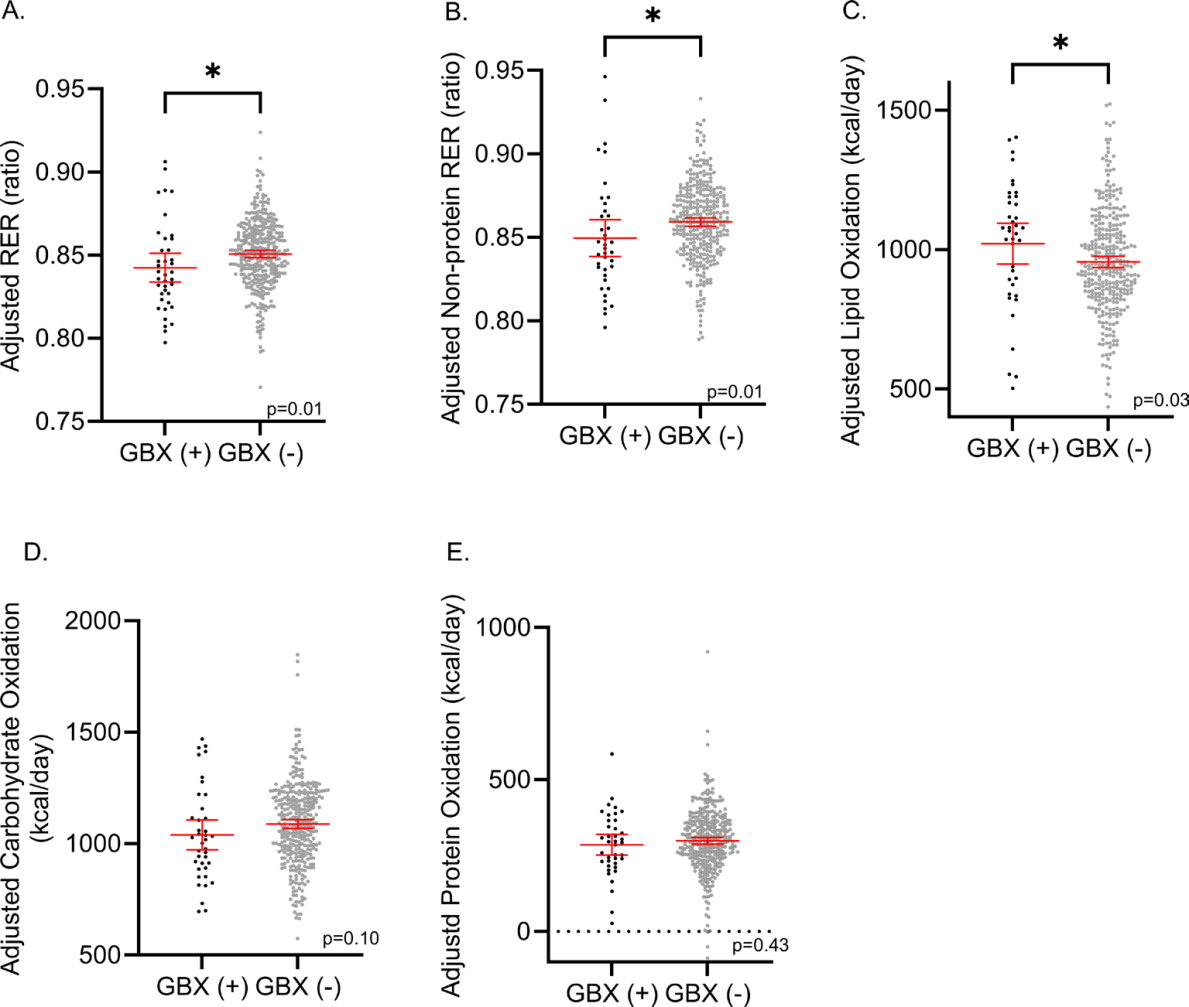
Differences in (A) respiratory exchange ratio (RER), (B) non‐protein RER, (C) lipid oxidation, (D) carbohydrate oxidation, (E) and protein oxidation between those with and without prior cholecystectomy (GBX). RER and non‐protein RER were lower, and lipid oxidation was higher in the prior cholecystectomy group. Models were adjusted for age, sex, body composition, impaired glucose regulation (vs. normal glucose regulation), and energy balance during the eucaloric chamber. Mean values were added back to residuals to restore the original scale. Asterisk (*) denotes statistical significance (*p* < 0.05). [Color figure can be viewed at wileyonlinelibrary.com]

RER (*β* = −0.009 ratio, SE = 0.004, *p* = 0.02), non‐protein RER (*β* = −0.01 ratio, SE = 0.005, *p* = 0.02), and CarbOx rate (*β* = −137 kcal/day, SE = 39.64, *p* = 0.0006) were lower in GBX(+) (Table 2). In models adjusted for covariates, RER and non‐protein RER remained lower (*β* = −0.01 ratio, SE = 0.004, *p* = 0.01; *β* = −0.01 ratio, SE = 0.005, *p* = 0.01; Figure 2A,B, respectively) reflected in higher LipOx (*β* = 79 kcal/day, SE = 35.61, *p* = 0.03, Figure 2C) in the GBX(+) group. CarbOx (*β* = −59 kcal/day, SE = 35.32, *p* = 0.10) and ProtOx (*β* = −15 kcal/day, SE = 19.71, *p* = 0.43) rates were not significantly different by GBX status in adjusted models (Figure 2D,E). In separate sensitivity analyses in the female subgroup and in those with normal glucose regulation, results were similar (Figures S5 and S6).

### 3.4 Bile Acids

BA measurements were available for 61 participants (all female), including 18 with and 43 without prior GBX (Table S2). Conjugated primary BAs glycocholic acid (GCA) and glycochenodeoxycholic acid (GCDCA), along with the conjugated secondary BA glycoursodeoxycholic acid (GUDCA), were higher in GBX(+) (*β* = 0.3 ng/mL, SE = 0.11, *p* = 0.011; *β* = 0.27 ng/mL, SE = 0.10, *p* = 0.01; *β* = 0.21 ng/mL, SE = 0.10, *p* = 0.048, respectively). Concentrations of other BAs did not differ between groups (Table S2). Analyses using general linear models were conducted to examine the potential effects of BAs that differed between GBX(+) and GBX(−) groups on EE variables, but no associations were observed (data not shown).

## 4 Discussion

The current study investigated the relationship between prior GBX and energy metabolism focusing on EE, RER, and substrate oxidation. Our results demonstrated that participants with a history of GBX [GBX(+)] had higher sleep EE, along with lower total RER and non‐protein RER, compared to those without GBX. The lower RER observed in the GBX(+) group was reflected in higher LipOx.

Although GBX is a safe and necessary procedure, its long‐term consequences on energy metabolism are unclear. That sleep EE was higher in the GBX(+) group is consistent with the limited prior studies in animals and humans. Compared with sham‐operated mice, gallbladder ablation increased basal metabolic rate, which is akin to sleep EE in our study [13]. In humans, resting EE was increased in 40 persons after laparoscopic GBX compared with 37 control patients [31]. However, fat utilization was lower after GBX rather than higher as was the case in our study. The reason for this difference is unclear and may be due to racial or ethnic differences or surgical indications (gallbladder polyps in Yin et al. study [31]). The discrepancy between studies might also relate to the impact of prior diet on RER in the Yin study [31]. Because RER is affected by diet, our unit and other labs use a “wash out” period of approximately 3 to 5 days where participants receive a standard WMEN. In contrast, there was no standard diet in the Yin study [31], with RER assessed after a 12‐h fast. This may account for some of the differences we observe here. The precise mechanisms by which GBX leads to higher sleep EE or higher lipid utilization in the current study are uncertain but are possibly due to altered BA secretion and circulation. Prior evidence suggests that BAs may have an impact on energy metabolism. The gallbladder regulates the rhythmic secretion of BAs, which not only facilitate lipid digestion but also contribute to EE and metabolic homeostasis. This occurs through signaling pathways involving the TGR5 [9]. Removal of the gallbladder can cause change in these pathways but also increased enterohepatic circulation of BAs [8]. Both transport of BAs across the sinusoidal membrane of the liver and reabsorption in the lumen of the small intestine occur by energy dependent processes [13], which could explain the higher sleep EE in the GBX(+) group.

BAs activate the intestinal TGR5 pathway, exerting further metabolic effects. The TGR5 pathway increased EE via increasing 3,5,3′‐triiodothyronine (T3) levels in brown adipose tissue and skeletal muscle. Thus, an effect of GBX on the TGR5 pathway may also affect energy metabolism and substrate oxidation [32]. The gut microbiome is also altered after GBX [33]. The gut microbiome is known to impact energy metabolism in animal models [34]. As noted earlier, several BAs (GCA, GCDCA, UDCA) were higher in the GBX(+) group but were not associated with energy metabolism measures including sleep EE, RER, and LipOx. It is possible that the relatively small subset of participants for whom BA measurements were available reduced our power to observe effects of BAs on these measures. Sleep EE was elevated in those with GBX, but no difference in 24‐h EE was observed between groups. The inability to detect a difference in 24‐h EE may be due to insufficient size or higher within‐subjects variability in EE during the awake period compared with sleep EE [35].

A key strength of this study is the use of 24‐h whole room indirect calorimetry, which allowed for detailed assessment of the various components of EE. However, the findings should be interpreted considering certain limitations. First, the study population was composed of Indigenous American participants, and most individuals who had undergone GBX were women. As a result, the generalizability of these findings to other populations may be limited. As there was a sex imbalance, sensitivity analyses and sex‐ and age‐matched analyses, where differences in physical characteristics were not significant, yield similar findings to the main results. Second, the cross‐sectional design means that measurements were collected at a single time point, making it difficult to account for temporal fluctuations. Third, habitual food intake may influence RER measured in the respiratory chamber. Although participants were provided a standardized diet (food quotient 0.87) on the metabolic ward for 5 days prior to the respiratory chamber stay [18], it is unknown if there were any differences in food quotient between cases and controls prior to the inpatient stay. The present analyses were performed on cohorts that had starting dates of 1985 and 2007. Differences in assessment years were not examined and may potentially bias or attenuate results.

## 5 Conclusion

The current study shows increased sleep EE, LipOx, and decreased RER after GBX. We demonstrated altered concentrations of some BAs in a subset of participants but no association with these and our EE or substrate oxidation differences. Thus, despite its effect on BA metabolism, gallbladder removal may have an effect on metabolic fuel selection and basal metabolic rate.

## Author Contributions

**Beyza N. Aydin:** conceptualization, methodology, formal analysis, investigation, writing – original draft, visualization. **Emma J. Stinson**, **Tomás Cabeza de Baca**, **Helen C. Looker:** conceptualization, methodology, formal analysis, investigation, writing – review and editing. **Peter Walter:** investigation, writing – review and editing. **Jonathan Krakoff** and **Douglas C. Chang:** conceptualization, methodology, formal analysis, investigation, writing – review and editing, visualization, supervision. **Douglas C. Chang:** guarantor of the work and, as such, had full access to all the study data and takes responsibility for the integrity of the data and the accuracy of the data analysis.

## Funding

This research was supported by the Intramural Research Program of the National Institutes of Health, National Institute of Diabetes and Digestive and Kidney Diseases (grant number DK069015‐36 and DK069028).

## Conflicts of Interest

The authors declare no conflicts of interest.

## Supporting information


**Figure S1:** oby70145‐sup‐0001‐FigureS1.pdf.

**Figure S2:** The figure plots the association between fat‐free mass (%) and adjusted sleep energy expenditure (kcal/day). Sleep energy expenditure was adjusted for age, sex, body composition, impaired glucose regulation (vs. normal glucose regulation) and energy balance during the eucaloric chamber. Mean values were added back to residuals to restore the original scale.

**Figure S3:** Differences in (A) 24‐h energy expenditure (EE), (B) sleep EE, (C) awake and fed thermogenesis (AFT), (D) inactive state EE and (E) spontaneous physical activity (SPA) between those with and without prior cholecystectomy (GBX) in female subgroup (*n* = 162). Models were adjusted for age, body composition, and impaired glucose regulation (vs. normal glucose regulation). Mean values were added back to residuals to restore the original scale.

**Figure S4:** Differences in (A) 24‐h energy expenditure (EE), (B) sleep EE, (C) awake and fed thermogenesis (AFT), (D) inactive state EE, and (E) spontaneous physical activity (SPA) between those with and without prior cholecystectomy (GBX) in participants who had normal glucose regulation subgroup (*n* = 254). Models were adjusted for age, sex, body composition, and impaired glucose regulation (vs. normal glucose regulation). Mean values were added back to residuals to restore the original scale.

**Figure S5:** Differences in (A) respiratory exchange ratio (RER), (B) non‐protein RER, (C) lipid oxidation, (D) carbohydrate oxidation, and (E) protein oxidation between those with and without prior cholecystectomy (GBX) in female subgroup (*n* = 162). Models were adjusted for age, body composition, impaired glucose regulation (vs. normal glucose regulation) and energy balance during the eucaloric chamber. Mean values were added back to residuals to restore the original scale.

**Figure S6:** Differences in (A) respiratory exchange ratio (RER), (B) non‐protein RER, (C) lipid oxidation, (D) carbohydrate oxidation, and (E) protein oxidation between those with and without prior cholecystectomy (GBX) in participants who had normal glucose regulation subgroup (*n* = 254). Models were adjusted for age, sex, body composition, impaired glucose regulation (vs. normal glucose regulation) and energy balance during the eucaloric chamber. Mean values were added back to residuals to restore the original scale.

**Table S1:** Characteristics of female participants with (+) and without (−) prior GBX.
**Table S2:** Bile acid results of participants with (+) and without (−) prior GBX.

**Table S3:** Age and sex matched analyses.

## Data Availability

Deidentified individual participant data analyzed during this study will be made available from the corresponding author upon reasonable request pending application and approval.
