# Supplementary figures and images for: Cholecystectomy Is Linked With Lower Respiratory Exchange Ratio and Higher Lipid Oxidation and Sleep Energy Expenditure
Source: Obesity (Silver Spring). 2026 Feb 12;34(4):793–800. doi: 10.1002/oby.70145 (PMC13032049; doi:10.1002/oby.70145)

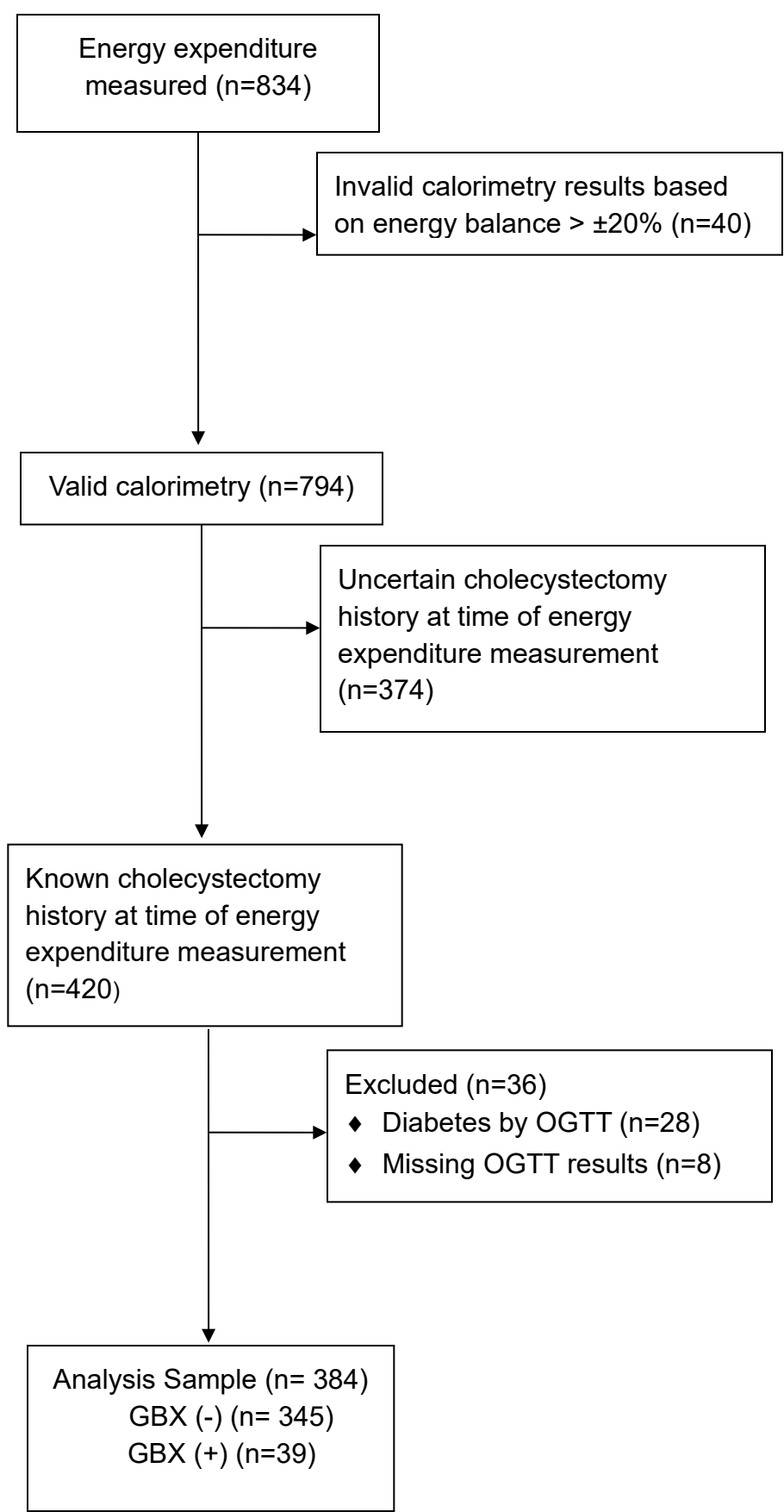

Supplement: Supplementary file 1 — Figure S1: oby70145‐sup‐0001‐FigureS1.pdf. [file OBY-34-793-s003.pdf]

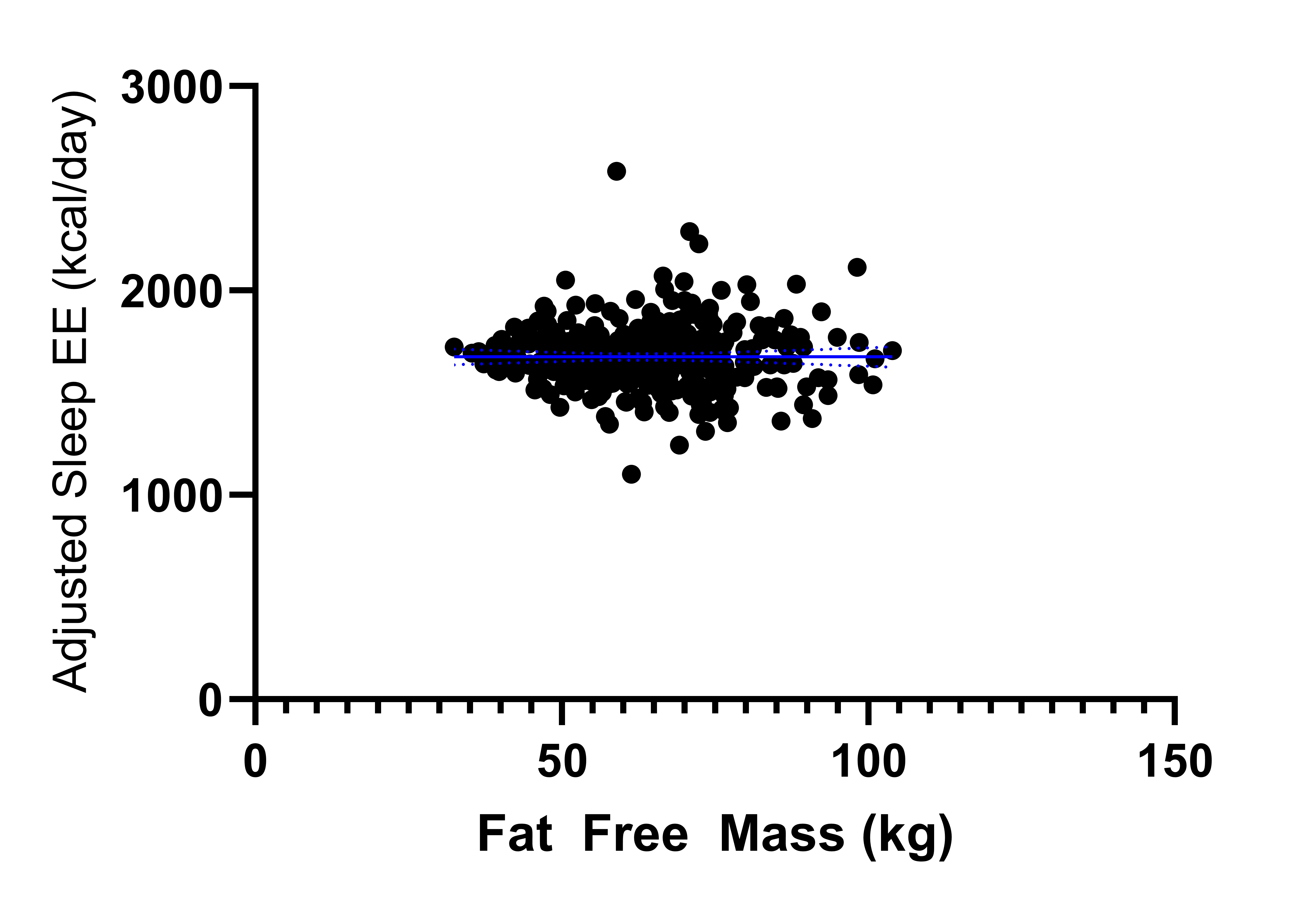

Supplement: Supplementary file 2 — Figure S2: The figure plots the association between fat‐free mass (%) and adjusted sleep energy expenditure (kcal/day). Sleep energy expenditure was adjusted for age, sex, body composition, impaired glucose regulation (vs. normal glucose regulation) and energy balance during the eucaloric chamber. Mean values were added back to residuals to restore the original scale. [file OBY-34-793-s004.tif]
