# Supplementary material for: Cholecystectomy Is Linked With Lower Respiratory Exchange Ratio and Higher Lipid Oxidation and Sleep Energy Expenditure
Source: Obesity (Silver Spring). 2026 Feb 12;34(4):793–800. doi: 10.1002/oby.70145 (PMC13032049; doi:10.1002/oby.70145)
Supplement: Supplementary file 7 — Table S1: Characteristics of female participants with (+) and without (−) prior GBX. Table S2: Bile acid results of participants with (+) and without (−) prior GBX. [file OBY-34-793-s006.docx]

| **Supplementary Table 1. Characteristics of female participants with (+) and without (-) prior GBX** | | | | |
| --- | --- | --- | --- | --- |
| **Variable** | **Total** | **GBX (+)** | **GBX (-)** | **P value** |
| Female, n (%) | 162 (100) | 37 (23) | 125 (77) | **-** |
| Age (year) * | 28.1 ± 6.2 | 31.2 ± 6.5 | 27.2 ± 5.8 | 0.0004 |
| **Body Composition** |  |  |  |  |
| Weight (kg) | 92.3 ± 26.3 | 97.4 ± 27.9 | 90.7 ± 25.7 | 0.2 |
| Body mass index (kg/m^2^) | 36.1 ± 9.4 | 38.1 ± 9.7 | 35.5 ± 9.3 | 0.1 |
| Body Fat (%) | 38.6 ± 6.2 | 40.0 ± 5.1 | 38.2 ±6.5 | 0.1 |
| Fat mass (kg) | 36.85 ± 15.2 | 40.0 ± 16.3 | 35.9 ± 14.8 | 0.2 |
| Fat-free mass (kg) | 55.4 ± 11.8 | 57.4 ± 12.1 | 54.8 ± 11.7 | 0.3 |
| **Oral glucose tolerance test** |  |  |  |  |
| Fasting plasma glucose (mg/dL) ***** | 91.1 ± 9.7 | 94.0 ± 10.0 | 90.3 ± 9.4 | 0.04 |
| 2-h plasma glucose (mg/dL) * | 131.6 ± 29.7 | 143.5 ± 27.3 | 128.0 ± 29.5 | 0.005 |
| Glucose regulation status, n (%) |  |  |  | 0.1 |
| Normal glucose regulation | 85 (52) | 15 (9) | 70 (43) |  |
| Impaired glucose regulation | 77 (48) | 22 (14) | 55 (34) |  |
| *Values are expressed as means ± SD or n (%). GBX, cholecystectomy; p-values for differences between GBX (+) and GBX (-) by t-test or chi-square test where appropriate. *p<0.05* | | | | |

| **Supplementary Table 2. Bile acid results of participants with (+) and without (-) prior GBX** | | | | |
| --- | --- | --- | --- | --- |
| **Variable** | **All** n=61 | **GBX (-)** n=43 | **GBX (+)** n=18 | **p value** |
| **Primary bile acids** |  |  |  |  |
| Cholic acid, (CA, ng/ml) | 16 [10-29] | 16 [10-29] | 17 [9-30] | 0.7 |
| Chenodeoxycholic acid (CDCA, ng/ml) | 41 [19-100] | 34 [16-85] | 45 [41-168] | 0.2 |
| Glycocholic acid (GCA, ng/ml) * | 76 [46-198] | 61 [35-168] | 182 [65-297] | 0.02 |
| Taurocholic acid (TCA, ng/ml) | 16 [8-42] | 14 [7-41] | 25 [13-47] | 0.1 |
| Glycochenodeoxycholic acid (GCDCA, ng/ml) * | 359 [19-867] | 276 [176-665] | 635 [390-1038] | 0.01 |
| Taurochenodeoxycholic acid (TCDCA, ng/ml) | 49 [28-112] | 46 [25-109] | 71 [31-156] | 0.2 |
| **Secondary bile acids** |  |  |  |  |
| Deoxycholic acid (DCA, ng/ml) | 133 [63-174] | 127 [46-174] | 139 [73-174] | 0.9 |
| Lithocholic acid (LCA, ng/ml) | 8 [5-11] | 8 [5-12] | 8 [6-10] | 0.7 |
| Ursodeoxycholic acid (UDCA, ng/ml) | 7 [2-12] | 6 [2-11] | 8 [4-20] | 0.7 |
| Glycodeoxycholic acid (GDCA, ng/ml) | 207 [115-315] | 158 [103-274] | 276 [219-559] | 0.5 |
| Taurodeoxycholic acid (TDCA, ng/ml) | 27 [15-70] | 22 [12-63] | 47 [22-87] | 0.7 |
| Hyodeoxycholic acid (HDCA, ng/ml) | 12 [7-18] | 12 [8-18] | 12 [7-19] | 0.6 |
| Taurolithocholic acid (TLCA, ng/ml) | 1.9 [0.6-4.1] | 1.2 [0.5-4.1] | 2.8 [0.9-4.8] | 0.3 |
| Sulfated taurolithocholic acid (TLCA-3-sulfate, ng/ml) | 54 [31-110] | 54 [30-129] | 57 [31-102] | 0.5 |
| Glycoursodeoxycholic acid (GUDCA, ng/ml) * | 23 [16-49] | 21 [12-41] | 31 [22-72] | 0.048 |
| Glycohyodeoxycholic acid (GHCA, ng/ml) | 8 [5-13] | 6 [4-10] | 8 [7-15] | 0.6 |
| *Values are expressed as medians [1^st^ and 3^rd^ quartile]. GBX; cholecystectomy. Variables are not normally distributed; thus, p-values derived from t-tests on log-transformed values are reported. *p<0.05* | | | | |
