# Supplementary material for: Cholecystectomy Is Linked With Lower Respiratory Exchange Ratio and Higher Lipid Oxidation and Sleep Energy Expenditure
Source: Obesity (Silver Spring). 2026 Feb 12;34(4):793–800. doi: 10.1002/oby.70145 (PMC13032049; doi:10.1002/oby.70145)
Supplement: Supplementary file 8 — Table S3: Age and sex matched analyses. [file OBY-34-793-s007.docx]

1. ***Matched Analysis***

A matched analysis was performed whereby the 39 GBX(+) cases were age- and sex-matched by one GBX(-) control (1). Matching SAS macro (1) was set to select GBX(-) controls that were +/- 2 years from the respective case. No significant differences were found between the GBX(+) cases and GBX(-) matched controls on age (*p* = 0.84), BMI (*p* = 0.24), fat mass (*p* = 0.23), fat free mass (*p* = 0.19), and glucose impaired status (*p* = 0.26). Conditional logistic regression (using case-control status as an outcome) and general linear models (using case-control status as a predictor and similar to our original analyses) were performed. **Supplementary Table 3** presents the p-values for both sets of analyses. Consistent with the main analyses, when compared to controls GBX(-), GBX(+) cases had significantly higher 24-h sleep EE (exact β= 0.002 kcal/day, p=0.0083; β = 160.45 kcal/day, p=0.0299), and higher inactive state EE (exact β= 0.003 kcal/day, p=0.0308; β = 103.58 kcal/day, p=0.1047 [GLM was not significant]). Respiratory exchange ratio and substrate oxidation results were also consistent with the main analyses. When compared to controls GBX(-), GBX(+) cases had lower RER (exact β= -23.43 ratio, p= 0.0119; β = -0.014 ratio, p=0.0223), lower non-protein RER (exact β= -20.67 ratio, p=0.0093; β = -0.015 ratio, p=0.052 [GLM was not significant]), and higher LipOx (exact β= 0.002 kcal/day, p=0.0199; β = 138.99 kcal/day, p=0.0577 [GLM was not significant]).

1. Mortensen LQ, Andresen K, Burcharth J, Pommergaard HC, Rosenberg J. Matching Cases and Controls Using SAS(R) Software. *Front Big Data* 2019;**2:** 4.

| **Supplementary Table 3. Age and Sex Matched Analyses** | | | | | | |
| --- | --- | --- | --- | --- | --- | --- |
|  | Conditional Logistic Regression | | | General Linear Models | | |
|  | Exact β | *SE* | *p* | β | *SE* | *p* |
| 24-h EE, kcal/day | 0.001 | 0.001 | 0.1694 | 117.33 | 91.87 | 0.2054 |
| 24-h Sleep EE, kcal/day | 0.002 | 0.001 | **0.0083** | 160.45 | 72.47 | **0.0299** |
| AFT, kcal/15-h | 0.001 | 0.003 | 0.7489 | 8.83 | 28.35 | 0.7566 |
| Inactive State EE, kcal/15-h | 0.003 | 0.002 | **0.0308** | 103.58 | 62.83 | 0.1047 |
| SPA, % | 0.051 | 0.11 | 0.6549 | 0.063 | 0.62 | 0.9192 |
| RER, Ratio | -23.43 | 10.23 | **0.0119** | -0.014 | 0.006 | **0.0223** |
| Non-Protein RER, Ratio | -20.67 | 8.81 | **0.0093** | -0.015 | 0.007 | 0.052 |
| LipOx, kcal/day | 0.002 | 0.001 | **0.0199** | 138.99 | 72.10 | 0.0577 |
| CarbOx, kcal/day | -0.001 | 0.001 | 0.5365 | -21.92 | 53.26 | 0.6818 |
| ProTox, kcal/day | -0.001 | 0.002 | 0.7461 | -0.208 | 28.77 | 0.9942 |
| Bolded p-values denote statistical significance (*p* < 0.05)  Exact β = Unlogged conditional logistic regression beta coefficient on a logit scale (prior to exponentiation)  β - unstandardized parameter estimate from General Linear Model  GBX(+) was outcome for Conditional logistic regression  GBX(-) Control was reference group for General Linear Models. | | | | | | |
